# Physiological characterization of secondary metabolite producing *Penicillium* cell factories

**DOI:** 10.1186/s40694-017-0036-z

**Published:** 2017-10-17

**Authors:** Sietske Grijseels, Jens Christian Nielsen, Jens Nielsen, Thomas Ostenfeld Larsen, Jens Christian Frisvad, Kristian Fog Nielsen, Rasmus John Normand Frandsen, Mhairi Workman

**Affiliations:** 1 Department of Biotechnology and Biomedicine, Technical University of Denmark, 2800 Kgs. Lyngby, Denmark; 2 Department of Biology and Biological Engineering, Chalmers University of Technology, 412 96 Gothenburg, Sweden; 3 Novo Nordisk Foundation Center for Biosustainability, Technical University of Denmark, 2800 Kgs. Lyngby, Denmark

**Keywords:** *Penicillium*, Submerged fermentation, Physiology, Secondary metabolite, Cell factory

## Abstract

**Background:**

*Penicillium* species are important producers of bioactive secondary metabolites. However, the immense diversity of the fungal kingdom is only scarcely represented in industrial bioprocesses and the upscaling of compound production remains a costly and labor intensive challenge. In order to facilitate the development of novel secondary metabolite producing processes, two routes are typically explored: optimization of the native producer or transferring the enzymatic pathway into a heterologous host. Recent genome sequencing of ten *Penicillium* species showed the vast amount of secondary metabolite gene clusters present in their genomes, and makes them accessible for rational strain improvement. In this study, we aimed to characterize the potential of these ten *Penicillium* species as native producing cell factories by testing their growth performance and secondary metabolite production in submerged cultivations.

**Results:**

Cultivation of the fungal species in controlled submerged bioreactors showed that the ten wild type *Penicillium* species had promising, highly reproducible growth characteristics in two different media. Analysis of the secondary metabolite production using liquid chromatography coupled with high resolution mass spectrometry proved that the species produced a broad range of secondary metabolites, at different stages of the fermentations. Metabolite profiling for identification of the known compounds resulted in identification of 34 metabolites; which included several with bioactive properties such as antibacterial, antifungal and anti-cancer activities. Additionally, several novel species–metabolite relationships were found.

**Conclusions:**

This study demonstrates that the fermentation characteristics and the highly reproducible performance in bioreactors of ten recently genome sequenced *Penicillium* species should be considered as very encouraging for the application of native hosts for production via submerged fermentation. The results are particularly promising for the potential development of the ten analysed *Penicillium* species for production of novel bioactive compounds via submerged fermentations.

**Electronic supplementary material:**

The online version of this article (doi:10.1186/s40694-017-0036-z) contains supplementary material, which is available to authorized users.

## Background

Filamentous fungi are important producers of secondary metabolites: low-molecular-weight compounds that often have bioactive properties. The genus *Penicillium* currently includes more than 354 accepted species [1] many of which are capable of producing a wide variety of secondary metabolites [2]. The most well-known secondary metabolite produced by *Penicillium* is the antibiotic penicillin, which was discovered by Fleming [3] and which is nowadays produced in large scale using *P. rubens*, following intense strain improvement programs aimed at increasing the titers. Other important pharmaceutical compounds produced by *Penicillium* species include the antifungal griseofulvin [4], the immunosuppressant mycophenolic acid [5] and the cholesterol lowering drug compactin/mevastatin [6–8]. These examples illustrate the great importance of *Penicillium* species as production hosts and sources of bioactive compounds with medical applications. On the other hand, *Penicillium* species can also produce mycotoxins such as citrinin, ochratoxin and patulin [2], which can pose a health risk to humans and animals.

The increasing prevalence of antibiotic resistance among pathogenic bacteria was in 2016 named by the UN General Assembly to be amongst the greatest and most urgent global risk factor for the health of humans [9]. However, the discovery of novel antibiotics has stagnated since the 1970’s and only few novel classes of antibiotics have been discovered since then [10, 11]. This is most likely a consequence of the fact that drug development is time consuming and expensive, while market value and dominance for antibiotics is limited by the development of resistance. The broad metabolic diversity found within the fungal kingdom, however, continues to provide a rich source of novel drug leads, this coupled with advancements within process engineering and physiological characterization tools, provides a strong basis for exploiting the chemical and physiological diversity in fungi to establish novel fungal based bioprocesses which can meet the demands of modern society for novel antimicrobials and pharmaceuticals.

To facilitate the process from identification of a beneficial compound to the establishment of an industrially feasible bio-based production process, two major strategies can be followed; i.e. find and optimizing the best native producer or transfer the involved genes to an established heterologous host. The latter strategy is typically desirable if the native producer is not suitable for industrial fermentation processes or is genetically inaccessible. However, production of secondary metabolites in heterologous hosts is still in the developmental stage and expressing complex multistep pathways to commercially viable titers has proven highly challenging and typically requires substantial optimization [12]. The transfer of the given genetic trait in addition requires a thorough understanding of the given biosynthetic pathway and the involved genes. The other strategy, advancement of the native organism for the production of secondary metabolites, can be favourable as this eliminates the often tedious work of moving the biosynthetic pathway to a heterologous host. However, taming a uncharacterized filamentous fungus for industrial scale production is not trivial, as natural secondary metabolite yields are often low which combined with the complex interplay between physiology, physical environment and morphology often causes problems during fermentation such as nonhomogeneous mixing and nutrient limitation [13]. Therefore, in many instances, relatively unexploited fungi are not tested in submerged bioreactor cultivations, even though this would provide the necessary process performance data to evaluate whether to optimize the native producing organism or to transfer the pathway into a heterologous host.

The recent genome sequencing of ten *Penicillium* species [14, 15] has provided valuable novel insight into the rich diversity in secondary metabolite gene clusters present in these *Penicillium* species and has in addition, made the species amenable to rational strain improvement. In this study, we analyzed the potential of these ten recently genome sequenced *Penicillium* species as native producing cell factories. The species were cultivated in highly controlled bioreactors both in a defined medium that allowed for quantitative physiological evaluation and in a secondary metabolite inducing complex medium, to evaluate overall process performance, growth rates and reproducibility. Additionally, secondary metabolite profiles of the species during different time points in the fermentation were studied. To our knowledge, none of these species had been tested in a bioreactor before. This work demonstrates an implementation strategy for novel fungal cell factories based on highly controlled submerged bioreactors cultivations and quantification of key physiological parameters.

## Methods

### Organisms

The species used in this study were *P. coprophilum* (IBT31321), *P. nalgiovense* (IBT 13039), *P. polonicum* (IBT 4502), *P. antarcticum* (IBT31811), *P. vulpinum* (IBT 29486), *P. arizonense* (IBT 12289), *P. solitum* (IBT 29525), *P. decumbens* (IBT11843), *P. flavigenum* (IBT 14082), and *P. steckii* (IBT 24891). All strains are available from the IBT culture collection (Department of Biotechnology and Biomedicine, Technical University of Denmark).

### Submerged bioreactor batch cultivations

With the aim of investigating the growth characteristics of ten potential secondary metabolite producing *Penicillium* species, submerged bioreactor cultivations were performed with each species in two different media in biological triplicates.

#### Media

Czapek yeast autolysate (CYA) medium was used for spore propagation, containing per liter of demineralized water: 30 g sucrose, 5 g yeast extract, 3 g NaNO_3_, 1 g K_2_HPO_4_, 0.5 g MgSO_4_·7H_2_O, 0.5 g KCl, 0.01 g FeSO_4_·7H_2_O, 20 g agar and 1 mL trace metal solution containing 0.1 g/L ZnSO_4_·7H_2_O and 0.05 g/L CuSO_4_·5H_2_O. The pH was adjusted to 6.2 with NaOH prior to autoclaving. All chemicals applied were obtained from Sigma-Aldrich (Sigma-Aldrich).

Batch cultivations were performed in CY medium (CM) and in a defined medium (DM) for *Penicillium.* The defined medium contained per liter of demineralized water: 15 g glucose, 3.5 g (NH_4_)_2_SO_4_, 0.5 g MgSO_4_·7H_2_O, 0.15 g Na_2_-EDTA, 0.04 g FeSO_4_·7H_2_O, 0.8 g KH_2_PO_4_, 5 mL trace solution containing 1 g/L CuSO_4_·5H_2_O, 4 g/L ZnSO_4_·7H_2_O, 4 g/L MnSO_4_·7H_2_O, 1 g/L CaCl_2_·H_2_O. The CY medium contained the same components as stated above for spore propagation without the agar and 15 g sucrose per liter. The medium was heat sterilized by autoclaving at 121 °C for 20 min. After media sterilization, a sucrose solution (for CM) or glucose solution (for DM), separately autoclaved at 121 °C, was added.

#### Preparation of spore inoculum

Spores were propagated on CYA plates for 7 days and harvested with approximately 5 mL of cold MiliQ water containing 0.9% NaCl and 0.01% Tween solution, followed by filtration through Miracloth (Merck Millipore), centrifuged at 5000 g and the spore pellet washed again with 10 mL MiliQ water. Spores were counted using a Bürker–Türk counting chamber, followed by inoculation of bioreactors to a final spore concentration of 1 × 10^9^ spores/L.

#### Bioreactors

All bioreactor batch cultivations were carried out in Sartorious 1 L bioreactors (Satorious Stedim Biotech) with a working volume of 0.9 L, equipped with 2 Rushton six-blade disc turbines. Throughout the cultivation the temperature was maintained at 25 °C and the pH was kept constant at 6.5 by automatic addition of 2 M NaOH or H_2_SO_4_. The bioreactors were sparged with sterile atmospheric air. For the first 1200 min, the airflow was linearly increased from 0.1 volume of air per volume of liquid per minute (vvm) to 0.9 vvm and the stirring rate from 100 to 600 rpm. After that, these parameters were kept constant. The off-gas concentrations of oxygen and carbon dioxide were measured with a Prima Pro Process Mass Spectrometer (Thermo-Fischer Scientific), calibrated monthly with gas mixtures containing 5% (v/v) CO_2_, 0.04% (v/v) ethanol and methanol, 1% (v/v) argon, 5% (v/v) and 15% (v/v) oxygen all with nitrogen as carrier gas. The pH electrode (Mettler Toledo) was calibrated according to manufacturer’s standard procedures.

#### Biomass dry weight determination

Growth rates were determined based on biomass dry weight samples collected during the exponential growth phase. First a known amount of cell culture (typically around 3 mL) was filtered through pre-dried and weighted 0.45 µm polyether sulfone filters (Sartorius Stedim Biotech) which was then washed with demineralized water. The filter was folded to lock the biomass inside and dried in a microwave at 150 W for 20 min. The dry weight was measured after a cooling period of approximately 30 min in a desiccator.

#### Glucose concentration determination

For quantification of the glucose concentration in the culture medium, fermentation broth was filtered through a Q-Max^®^ Ca-Plus Filter (Frisenette ApS) with a pore size of 0.45 µm and stored at −20 °C until analysis. Separation and detection of the compounds was accomplished with a high performance liquid chromatography (HPLC) system equipped with a Bio-Rad Aminex HPX-87H column (BioRad) coupled to a RI detector. Elution was performed isocratically with H_2_SO_4_ (5 mM) as the mobile phase with a flow velocity of 0.6 mL/min at 60 °C. Quantification was performed using a six-level external calibration curve.

### Calculations of physiological characteristics

The end of the lag phase was determined as the time point were the percentage of CO_2_ in the off gas was consecutively over 6%.

Maximum specific growth rates were calculated via linear regression on a semi-logarithmic scale on at least three experimental biomass dry weight data points during the exponential phase and all R^2^ values were at least 0.96. Maximum CO_2_ production rates where determined similarly but with an R^2^ of at least 0.99. A custom script was developed in the statistical programming language R to determine the duration of the non-exponential growth phase for all cultivations, defined as the time difference between the end of the exponential fit to the CO_2_ exhaust, and the time point where the maximum overall CO_2_ production was reached. The code is available at: https://github.com/JensChrNielsen/Fungal-Biol-Biotechnol-2017.

The overall biomass yield on glucose was calculated as the ratio between the biomass gain and the glucose consumption in the growth phase.

### Secondary metabolite analysis

With the aim of identifying the secondary metabolite profiles in the fermentation broth of the ten *Penicillium* species grown in two different media, the filtered broth was extracted with ethyl acetate, analyzed with ultra-high performance liquid chromatography-diode array detection-quadrupole time of flight mass spectrometry UHPLC–DAD–QTOFMS, and known compounds were identified using an in-house compound library.

#### Extraction

The fermentation broth was filtered through a 0.45 µm cellulose acetate filter (Frisenette), for extraction of non-polar compounds 1.0 mL of ethyl acetate extraction solvent was added to 0.8 mL of filtrate. After ultra-sonification for 1 h and centrifugation for 1 min at 13,000*g* the organic (top) layer was transferred to a clean vial. For extraction of polar compounds 1.0 mL of acidic ethyl acetate (ethyl acetate + 0.5% formic acid) was added to the remaining filtrate. Following ultra-sonification and centrifugation the organic (top) layer was added to the extraction solution containing the extracted non-polar compounds. The combined solution was evaporated to dryness and re-dissolved in 125 µL HPLC grade methanol. After centrifugation for 5 min at 13,000*g* the supernatant was directly used for chemical analysis.

#### UHPLC–DAD–QTOFMS analysis

Secondary metabolite analysis was achieved by UHPLC–DAD–QTOFMS on a maXis HD orthogonal acceleration quadrupole time-of-flight mass spectrometer (Bruker Daltonics) equipped with an electrospray ionization (ESI) source and coupled to an Ultimate 3000 UHPLC system (Dionex, Thermo Scientific). Separation was achieved with a Kinetex 2.6 µm C_18_, 100 × 2.1 mm (Phenomenex) column maintained at 40 °C with a flow rate of 0.4 mL/min. A linear gradient system composed of 20 mmol/L formic acid in water, and 20 mmol/L formic acid in acetonitrile was used, starting from 10% (v/v) acetonitrile and increased to 100% in 10 min, maintaining this rate for 3 min before returning to the starting conditions. The system was re-equilibrated before subsequent sample analysis. MS analysis was performed in ESI^+^ with a data acquisition rate of 10 scans per second at *m/z* 100–1000, switching between 0 and 20 eV fragmentation energy.

#### Identification of secondary metabolites

Identification of secondary metabolites was performed using aggressive dereplication of the full scan high resolution (HR) MS data, and pseudo MS/MS data from the 20 eV fragmentation trace, using a search list of compounds based on former taxonomic identification and a manual search of major peaks in the internal library. This library consists of 1500 compounds of which 95% are fungal secondary metabolites [16]. Compounds were confirmed by comparison of HRMS, UV/Vis and MS/HRMS to a reference standard. The number of peaks was counted by making a list of compounds (dereplicated and unknown ones) based on molecular features in the latest time sample in the fermentation, with an absolute area higher than 500,000 counts and intensity higher than 10,000 counts. Subsequently, the presence of these compounds was searched for in all the other samples. The total amount was determined by counting all compounds that were present in two or three of the triplicate samples, without using a threshold value.

## Results

### Physiology

**Fig. 1:**
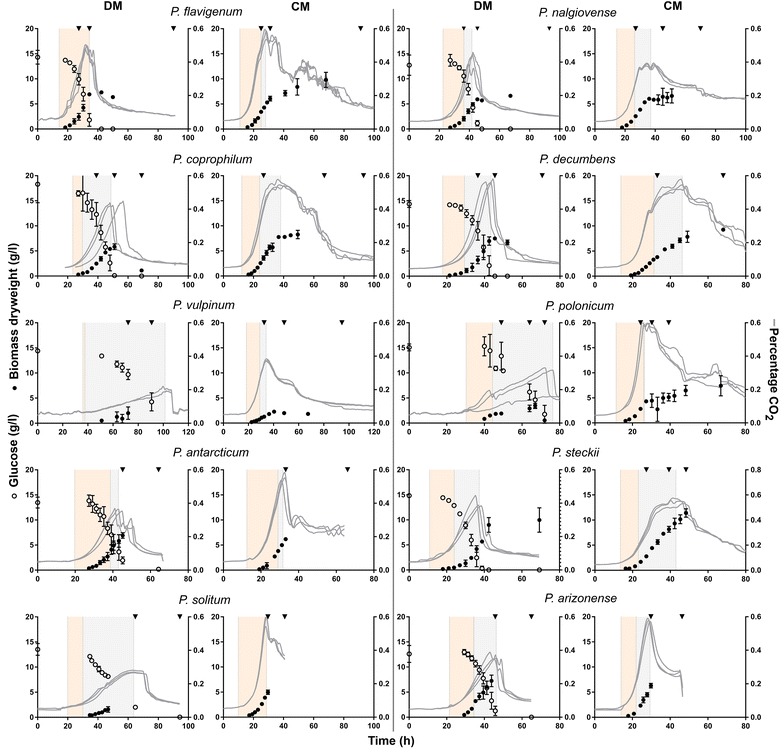
Fermentation profiles of ten *Penicillium* species cultivated in 1 L bioreactors in DM and CM. All fermentations were performed in biological triplicates. The CO_2_ exhaust values (%) are shown for all triplicate experiments separately as solid lines (right Y-axis), the dry weight (g/L) values are shown as a mean with standard deviations as black circles (left Y-axis) and for the defined medium the glucose concentrations are shown as mean with standard deviations as open circles (left Y-axis). Light orange shaded area shows the exponential phase and light grey shaded area shows the non-exponential phase. For *P. polonicum* one of the triplicate experiments showed a different growth curve, for this fermentation only the CO_2_ profile is shown. Triangles are the time points were samples for secondary metabolite analysis were taken. The third sample point in *P. decumbens* was at 120 h and falls therefore outside the figure. For *P. steckii* in DM the samples for secondary metabolite analysis were taken from a different experiment with similar growth characteristics

Ten different recently genome sequenced wild type *Penicillium* species were cultivated in 1 L controlled bioreactors to evaluate their behaviour in submerged cultivations. The species were grown in both a defined medium for *Penicillium* (DM) and in the more industrially relevant medium Czapek Yeast Autolysate (CM). During the course of the fermentations the CO_2_ off gas was continuously measured, while biomass dry weight was sampled until CO_2_ off gas values started declining. Additionally, for DM the glucose concentration was determined at regular time intervals throughout the fermentations. All cultivations were conducted in biological triplicates to evaluate the reproducibility of the experiments. The work revealed that the ten tested *Penicillium* species displayed highly diverse growth characteristics and responded differently to the two tested nutritional regimes, as evident from the growth curves (Fig. 1).

The initial lag phase, in which minimal changes in the CO_2_ levels were observed, lasted between 11 and 38 h on DM, while it was shorter for all species on CM lasting between 10 and 18 h, post inoculation (Table 1). In particular, *P. flavigenum* showed a short lag phase on both media where exponential growth was reached after 14 and 11 h for DM and CM, respectively. *Penicillium vulpinum* showed the longest lag phase in DM and CM of 38 and 18 h, respectively. Microscopic examination of culture broth from the ten species revealed that the lag phase was characterized by germination of spores followed by elongation of hyphae. After the lag phase, the exponential phase was initiated, where the growth rate reached its maximum. Here, the morphology of the ten species varied as a function of the species and media. In CM, all species initially grew as freely dispersed mycelium until the maximum CO_2_ off gas was reached, after which the formation of pellets typically started. In the DM, five of the ten species showed pellet formation relatively early in the fermentation; *P. steckii* and *P. solitum* showed the formation of small dense pellets, *P. vulpinum* and *P. coprophilum* showed a mixture of pellets and clumps, and *P. flavigenum* showed a mixture of small clumps and dispersed growth. The remaining species and all species in CM showed dispersed growth at least until the time of maximum CO_2_ off gas (Table 1).

**Table 1:** Physiological characteristics of ten *Penicillium* species cultivated in 1 L bioreactors in DM and CM

Species	Biomass yield growth phase (Y_sx_) DM (g DW/g glucose)	Lag phase DM (h)	Morphology DM	Lag phase CM (h)	Morphology CM
*P. flavigenum*	0.58 ± 0.05	14.3 ± 0.5	Small clumps/dispersed	10.9 ± 0.3	Dispersed
*P. nalgiovense*	0.60 ± 0.17	22.5 ± 0.8	Dispersed	14.5 ± 0.1	Dispersed
*P. coprophilum*	0.37 ± 0.03	23.3 ± 0.6	Pellet	12.2 ± 0.6	Dispersed
*P. decumbens*	0.55 ± 0.02	17.8 ± 0.9	Dispersed	13.8 ± 0.2	Dispersed
*P. vulpinum*	0.54 ± 0.14	37.6 ± 1.4	Pellet + clumps + wall	18.3 ± 0.2	Dispersed
*P. polonicum*	0.25 ± 0.01	30.3 ± 1.7	Wall growth	11.3 ± 0.1	Dispersed
*P. antarcticum*	0.58 ± 0.03	19.7 ± 0.2	Dispersed	12.5 ± 0.1	Dispersed
*P. steckii*	0.37 ± 0.02	10.9 ± 2.3	Pellet + wall growth	13.5 ± 0.2	Dispersed
*P. solitum*	0.29 ± 0.13	19.9 ± 1.2	Pellet + wall growth	9.9 ± 0.2	Dispersed
*P. arizonense*	0.67 ± 0.04	21.4 ± 0.4	Dispersed	14.0 ± 0.8	Dispersed

Biomass yield on glucose (g DW/g glucose), duration of lag phase (h) and observed morphology at the time point where the CO_2_ was at its maximum

**Fig. 2:**
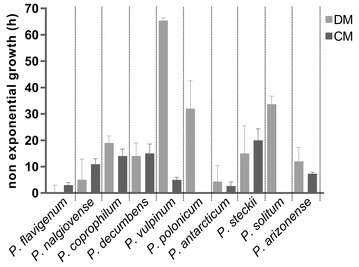
Length of non-exponential growth phase of ten *Penicillium* species cultivated in 1 L bioreactors in DM and CM. Non-exponential growth phase was the time in hours between the end of exponential growth phase and the maximum CO_2_ off gas value. Bars show average and standard deviation if triplicate submerged fermentations. *P. flavigenum* in DM, *P. polonicum* in CM and *P. solitum* in CM showed exponential growth until the maximum CO_2_ off gas value

The duration of the exponential phase proved to be correlated with the observed growth morphology, where the exponential growth transitioned into non-exponential growth at the same time as pellet or clump formation occurred. For seven of the ten species, the non-exponential growth phase was more than ten h in DM, while this was the case for four of ten species in the CM (Fig. 2). Only *P. flavigenum* and *P. antarcticum* showed exponential growth and dispersed morphology during the entire growth phase in both media (Fig. 2 and Additional file 1). In contrast, *P. vulpinum* showed dispersed growth and grew exponentially during the entire growth phase in CM, while in DM it had pelleted morphology and grew exponentially until 36 h, where after the CO_2_ off gas values were still increasing, but in a non-exponential manner, until after 110 h maximum values of exhausted CO_2_ off gas were reached.

**Fig. 3:**
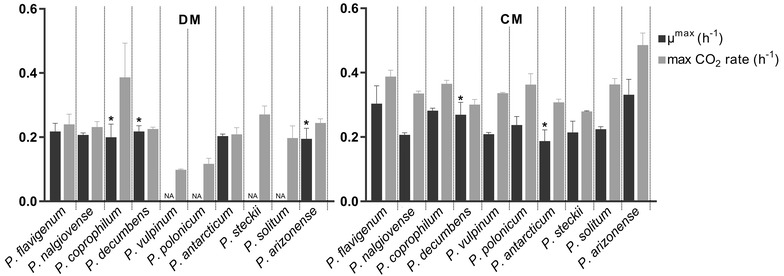
Maximum specific growth rates and maximum CO_2_ production rates for ten *Penicillium* species cultivated in 1 L bioreactors in DM and CM. Dark grey bars: maximum specific growth rates µ^max^ (h^−1^) and standard deviations calculated by linear regression of minimum three biomass dry weight data points during the exponential phase. For *P. vulpinum*, *P. polonicum*, *P. steckii*, and *P. solitum* in DM there were less than three dry weight samples during exponential phase and therefore µ^max^ could not be calculated. Light grey bars: maximum production rate of CO_2_ (h^−1^) and standard deviations calculated by linear regression of accumulated CO_2_ values during exponential growth. All values are based on independent triplicate fermentations, except for the ones with an asterix; there the values are based on duplicate fermentations

Maximum specific growth rates (µ^max^ **)** were calculated based on dry weight values during the exponential growth phase. For several species in DM it proved impossible to determine accurate growth rates based on biomass dry weight due to insufficient samples during the short exponential phase. As an alternative method for the growth rate, the rates of CO_2_ production were determined, as this is directly proportional to the amount of metabolically active biomass present, assuming that the yield and specific growth rate remain constant [17]. The species showed growth rates between 0.19 and 0.24 h^−1^ in DM and between 0.2 and 0.35 h^−1^ in CM (Fig. 3). The biomass yields on glucose during the growth phase (Y_sx_) varied highly among species; between 0.25 g DW/g glucose for *P. polonicum* to 0.67 g DW/g glucose for *P. arizonense*. The species with the lowest Y_sx_ values, *P. polonicum*, *P. steckii*, *P. solitum* and *P. coprophilum* (all under 0.4 g DW/g glucose) all showed pellet formation and/or wall growth, while species with high yields, *P. nalgiovense* and *P. arizonense* (0.6 and 0.67 g DW/g glucose), showed dispersed growth.

#### Secondary metabolite analysis

**Fig. 4:**
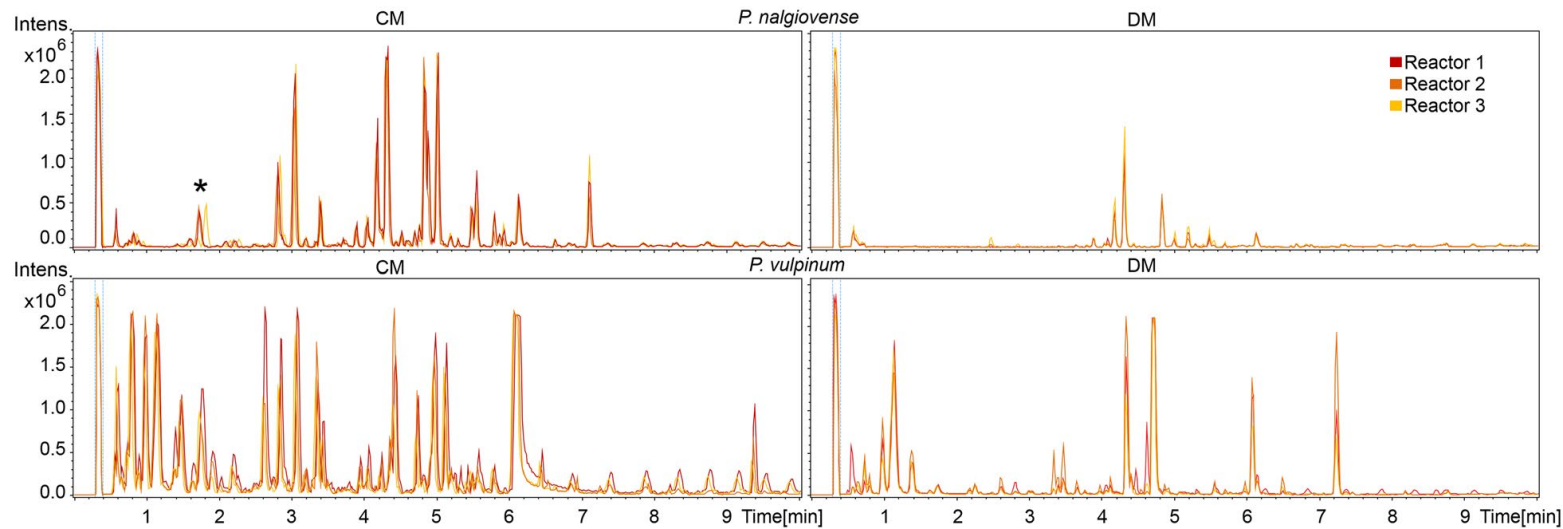
Base peak chromatograms (BPCs) of extracted samples of biological triplicates of *P. nalgiovense* and *P. vulpinum* fermentations in CM and DM. The BPCs of the triplicates are shown in red, dark orange and light orange. BPCs show peaks with the similar retention times and comparable peak areas. Peak with asterisk showed a slight change of retention times but have the same molecular features

Samples taken during different stages of the submerged cultivations were filtered, extracted, and analyzed for the presence of secondary metabolites in the fermentation medium using reverse phase UHPLC coupled to time-of-flight (QTOF) MS. Analysis of the biological triplicate experiments for each of the tested species in either of the two media were found to be highly reproducible based on the occurrence of base peak chromatogram (BPC) peaks with the same retention times and comparable peak areas in the normalized chromatograms (examples given in Fig. 4).

To allow for identification of the individual compounds found in the samples, the effluent from the UHPLC column was directly analysed by the attached QTOFMS which allowed for the recording of accurate mass. For each sample taken at the end of the cultivation the amount of molecular features (equivalent with the number of different compounds) above an area threshold of 500,000 and an intensity of 10,000 counts in the UHPLC–QTOFMS data was determined. The ten different species each displayed a unique metabolic profile in the two tested media (Table 2 and Additional file 2). A minimum of two compounds was observed in *P. flavigenum* cultivated in DM, and a maximum of 34 compounds in *P. steckii* cultivated in CM (last column in Table 2). In eight out of the ten species a greater number of compounds were detected in the cultivations conducted in CM compared to DM; exceptions were *P. polonicum*, where cultivation in DM showed a greater number of peaks than in CM, and *P. solitum*, where the number of compounds was equal in DM and CM.

**Table 2:**
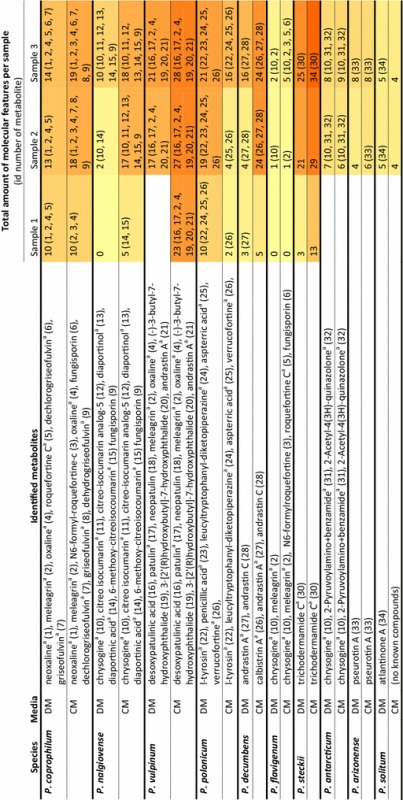
Metabolites produced in different growth phases of ten *Penicillium* species

Column 3: metabolites (and their id numbers) identified with dereplication, in order of retention time. Column 4, 5 and 6: per sample the total amount of molecular features (identified and unknown metabolites), in between brackets the id number of the identified compounds in each sample. A heat map from yellow to dark orange follows the total amount of molecular features in the samples

^a^Confirmed with a reference standard

Additionally, metabolite profiling was performed to identify the known compounds produced during the fermentations. In total 34 different metabolites were identified, from which most of them could be confirmed by comparison of HRMS, UV/Vis and MS/HRMS to a reference standard. Most compounds were unique to one of the ten species, with the exception of chrysogine identified in three species (*P. nalgiovense*, *P. flavigenum* and *P. antarcticum*), fungisporin in three species (*P. coprophilum*, *P. nalgiovense* and *P. flavigenum*) and andrastin A in two species (*P. vulpinum* and *P. decumbens*), while andrastin C was only identified in *P. decumbens*. Additionally, the roquefortine pathway was identified in three species (*P. coprophilum*, *P. vulpinum* and *P. flavigenum*), where depending on the media and species, different intermediates and end products of this pathway could be identified; in *P. flavigenum* only meleagrin and precursors were identified while both in *P. coprophilum* and *P. vulpinum* the end product of the pathway, oxaline was identified. Three species–metabolite relationships have, to our knowledge, not been described in literature before: *P. vulpinum* and *P. decumbens* producing andrastin A and *P. flavigenum* producing chrysogine.

To identify in which stages of the fermentation the various metabolites were produced, we examined the individual metabolite production profiles covering the three analyzed stages of the fermentation, one during the growth phase, one a few hours after the CO_2_ values started decreasing and one in the stationary phase (depicted as triangles in Fig. 1 and exact values in Additional file 2). This analysis revealed production of specific metabolites was associated with specific growth phases of the cultivations. For example for *P. coprophilum* in DM, the metabolites from the roquefortine/oxaline pathway were already present in the first sample, while griseofulvin and dechlorogriseofulvin could only be detected in the last sample. Another example of this stage depending production was *P. steckii* grown in CM where the vast majority of the metabolites were identified at the second sample point, while trichodermamide C was only first observed at the third sample point. Many of the metabolites in *P. steckii* were identified as belonging to the family of hynapenes and arohynapenes (based on UV and accurate mass) but because of a lack of reference standards could not be assigned to specific peaks and are therefore not shown in Table 2. In some cases, metabolites were produced only in CM or DM; penicillic acid was only produced in *P. polonicum* in CM, calbistrin A was only detected in *P. decumbens* in CM, and atlantinone A was only detected in *P. solitum* in DM. This was also seen for unidentified metabolites (Additional file 2). In 17 out of the 20 cultivations the metabolite levels in the third sample were higher than in the second sample, showing that part of the metabolites started to be produced only after the growth phase.

## Discussion

The fungal kingdom contains a huge reservoir of bioactive secondary metabolites; however, the development from discovery of a relevant metabolite to application remains a challenge. The recent genome sequencing of ten secondary metabolite rich *Penicillium* species facilitates exploitation of their genome sequences and/or their use as native producing cell factories. The aim of this study was to determine the suitability of these ten Penicillia as novel cell factories for native compounds by testing their growth performance and secondary metabolite production in submerged cultivations.

Cultivation of the fungal species in controlled submerged bioreactors proved that the ten wild type *Penicillium* species all could be cultivated using the same conditions. The cultivations proved to be highly reproducible, both in defined medium (DM) and rich medium (CM). In 18 out of the 20 cultivations, replicate values for CO_2_ off gas and biomass dry weight concentrations were reproducible with maximum CO_2_ production rates having standard deviations between 1 and 27%. The cultivations performed in CM showed dispersed growth until the maximum CO_2_ off gas was reached, while five out of the ten species cultivated in DM showed clump/pelleted growth. The fungal morphology can have an enormous impact on the production of enzymes and primary or secondary metabolites [18]. For example, micro-colonies are required for the production of citric acid by *A. niger* [19]. However, there is not a defined formula on how morphology affects productivity and it should be tested and optimized for the relevant species and metabolites. The work conducted in this study can be used as a starting point in optimization of process parameters for the production of a specific secondary metabolite.

Maximum specific growth rates in DM were calculated to be between 0.14 and 0.22 h^−1^ depending on the species, and in CM between 0.17 and 0.29 h^−1^. In case of pellet formation the cultures grew only exponentially in the beginning of the cultivation prior to pellet formation. The obtained growth rates are reasonable starting points for industrial fermentations, for example the penicillin producing *P. chrysogenum*, has a maximum growth rate under comparable conditions of around 0.2 h^−1^ [20, 21]. An interesting observation is that the species with the highest growth rates, *P. decumbens* and *P. coprophilum*, have the smallest genome size [15]. The biomass yields during the growth phase on glucose varied highly between species and there was a correlation between yields and morphology: species with low yields all showed pellet formation and/or wall growth, while species with high yields showed dispersed growth. In case of wall growth, this can be explained by the biomass determination method used in this study: broth was sampled from a port in the reactor, which in the case of wall growth resulted in a lower biomass value than the real biomass value in the reactor, and a lower yield. One hypothesis for the low yields observed in pelleted growth cultures is that dense hyphal packing may often result in diffusional limitation of both nutrients and oxygen, which can cause autolysis of the pellet core [22]. This can lead to a decrease of dry cell mass, as was previously observed in cultures of *P. chrysogenum* where hyphal elements showed cell wall degradation and clear signs of autolysis, even during the culture growth phase [23].

Similar to the growth profiles, the UHPLC–QTOFMS metabolite profiles showed to be reproducible in the biological triplicates of the batch fermentations. The base peak chromatograms revealed that all ten species produced secondary metabolites in submerged cultivations, with most of the metabolites being unique to one of the ten species. The media composition had an influence on secondary metabolite profiles, with eight out of ten species producing a higher number of compounds in CM than in DM. This is not surprising as the yeast extract containing CYA medium is regarded as a secondary metabolite inducing medium [24]. Some species such as *P. solitum* and *P. flavigenum* produced very few metabolites in both media; these species could be interesting for the development of secondary metabolite free production hosts, especially *P. flavigenum* since it showed to have a high reproducibility and a high growth rate. It has been shown in both *P. chrysogenum* [25] and in *Streptomyces* species [26, 27] that removal of highly producing secondary metabolite pathways can increase the production of other native, or heterologous secondary metabolites. However, the species producing very few secondary metabolites still contain many secondary metabolite biosynthetic gene clusters (BGCs). In a recent large scale genomic analysis of *Penicillium* genomes, it was found that 24 *Penicillium* genomes (including the ten used in this study) contained in total 1317 putative BGCs, corresponding to an average of 55 clusters per species [15]. This means that a great number of BGCs were inactive in the conditions used in this study, especially when taken into account that one BGC is often responsible for the biosynthesis of a whole family of related metabolites.

Dereplication of the extracts showed that the species produced a broad spectrum of secondary metabolites belonging to several different metabolite classes (polyketides, non-ribosomal peptides and terpenes). In total we identified 34 different known compounds, of which many for the first time in submerged fermentations. Here, it has to be taken into account that because of the chemical diversity of the secondary metabolites the extraction could not be optimal for all compounds, and it was only possible to obtain semi-quantitative data. Several of the identified compounds are known to have antibacterial, antifungal and anti-cancer activities, such as the antifungal griseofulvin, the antibiotic roquefortines [28] as well as the promising anti-cancer metabolites andrastin A [29, 30] and calbistrin A [31]. At the same time also several mycotoxins such as patulin and penicillic acid were produced of which elimination need to be considered in a production process. Additionally, we described three new species–metabolite relationships: to our knowledge, this is the first time that *P. decumbens* and *P. vulpinum* are described as andrastin A producers and *P. flavigenum* as chrysogine producer. Furthermore, it was shown that the species produce many unknown compounds at levels above the used detection area threshold; these could be interesting targets for isolation, bioactivity testing and structural elucidation, especially because it is less laborious to isolate metabolites from submerged cultures than from agar plates.

In order to utilize metabolic reprogramming techniques to optimize production of a secondary metabolite, the corresponding BGC of the metabolite must first be identified. The BGCs responsible for production of the detected andrastin A, patulin, griseofulvin, pseurotin A, fungisporin and roquefortine/meleagrin pathways have all previously been identified and characterized [32–42]. Although the experimental characterization was done in different species, represents for each of the listed BGCs, except for andrastin A, were recently identified in at least one of the ten analyzed species used in this study [15]. Several other metabolites with predicted BGCs and previously detected in solid cultivations of the corresponding strains, could not be detected in the submerged cultivations of the same strain. This was the case for yanuthones previously detected in extracts of *P. flavigenum* grown on agar plates [15], and fumagillin, pyripyropenes, austalides, and tryptoquivalines, previously detected in extracts of *P. arizonense* grown on agar plates [14]. In this context it is interesting that a species like *P. arizonense* only secreted pseurotin A, despite the large potential for producing a vast number of secondary metabolites. This would be an advantage if pseurotin A has to be produced in an industrial fermentation, but a disadvantage if any of its other secondary metabolites should be produced [14]. Furthermore we detected small amounts of penicillin G in the CM extracts of the known penicillin G producer *P. nalgiovense*, but the intensity and area were lower than the threshold values used in this study. It should be considered that in the experiments conducted in this study the biomass was filtered out and only the secreted metabolites were studied, while in plate experiments the intracellular metabolites are also taken into account.

## Conclusions

The results of this study show that the fermentation properties of the ten analysed *Penicillium* species and the highly reproducible performance in bioreactors should be considered as very encouraging for the application of native hosts for production via submerged fermentation. The *Penicillium* species (isolates) showed promising growth characteristics for use in large-scale industrial bioprocesses and produced a diverse range of interesting secondary metabolites. The production of specific secondary metabolites can subsequently be optimized by process optimization, classical strain development and/or with metabolic engineering approaches. Furthermore, all ten species used in this study were recently genome sequenced, which makes the use of rational approaches for improving the product yields or eliminating the production of other compounds more accessible thanks to the ongoing development of CRISPR/Cas9 genome editing techniques, which has recently been successfully demonstrated in filamentous fungi [43, 44]. The work conducted in this study can assist in deciding the optimization strategy for specific secondary metabolites produced by one of the ten *Penicillium* species.

## Additional files

**Additional file 1.** Physiological data. Physiological characteristics for triplicate fermentations of each species in DM and CM. Figures show the CO_2_ exhaust values and log value of the accumulated CO_2_. Furthermore a red line through the data points in exponential phase and highlighted in light grey the non-exponential phase; the time between start of non-exponential growth until the maximum CO_2_ off gas value. Additionally the R-squared value for the exponential phase, CO_2_ production rate, the number of data points in exponential phase, the end point (in hours) of the exponential phase and the end point of the non-exponential phase (in hours) are shown.

**Additional file 2.** Secondary metabolite data. In each separate tab one species with all triplicate samples taken in the DM and CM media. The time points of the samples taken (in hour after inoculation) and the metabolites detected with their maximum *m/z* value, retention time and intensity. The metabolites were first identified if they had an absolute area higher than 500,000 counts and intensity higher than 10,000 in the time last sample. The presence of these compounds was searched for in all the other samples. Data was generated by UHPLC–DAD–QTOFMS on a maXis HD orthogonal acceleration quadrupole time-of-flight mass spectrometer (Bruker Daltonics).
