# Supplementary figures and images for: Physiological characterization of secondary metabolite producing Penicillium cell factories
Source: Fungal Biol Biotechnol. 2017 Oct 17;4:8. doi: 10.1186/s40694-017-0036-z (PMC5644182; doi:10.1186/s40694-017-0036-z)

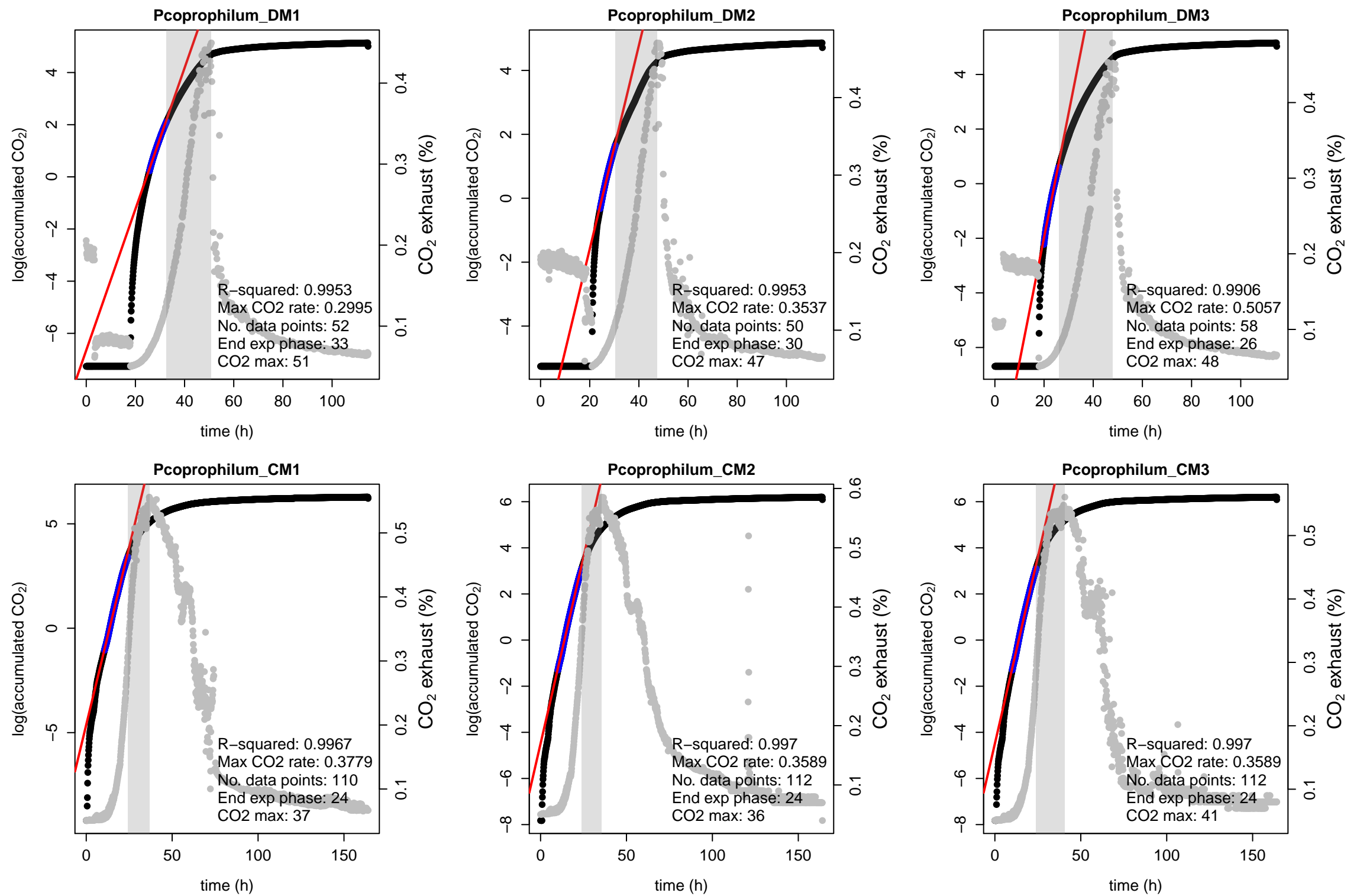

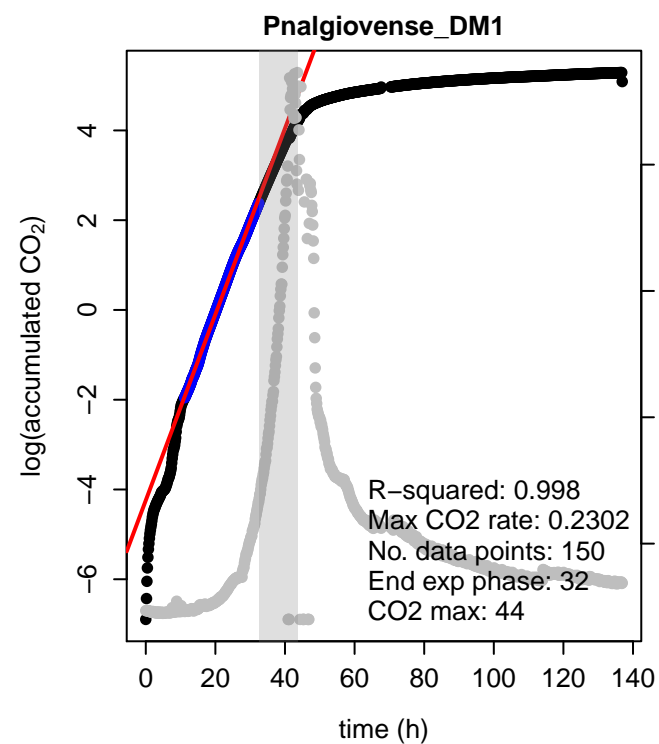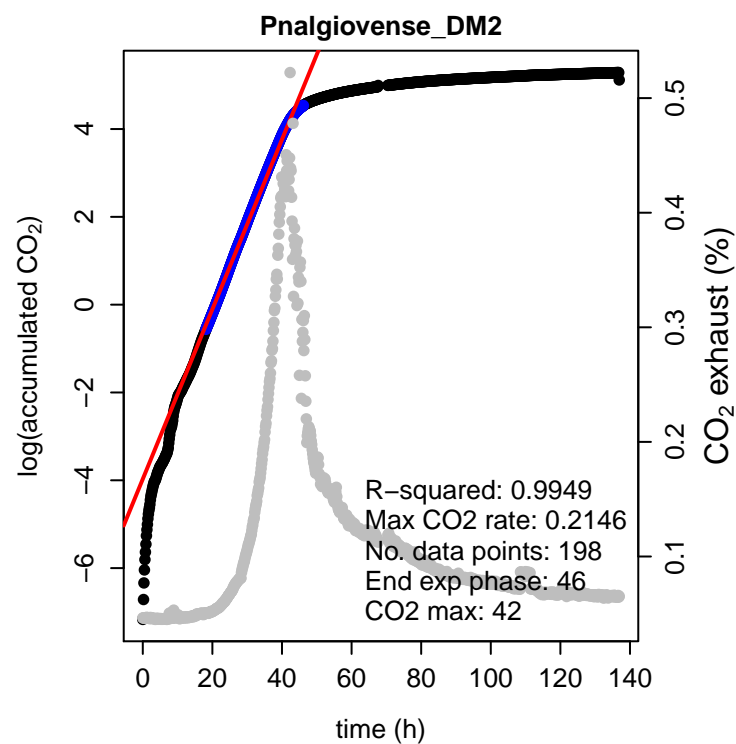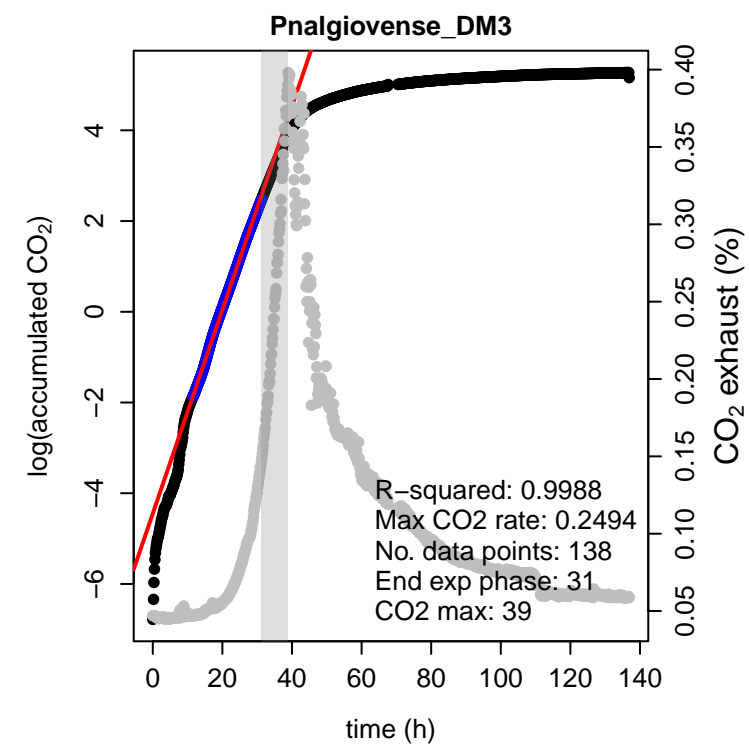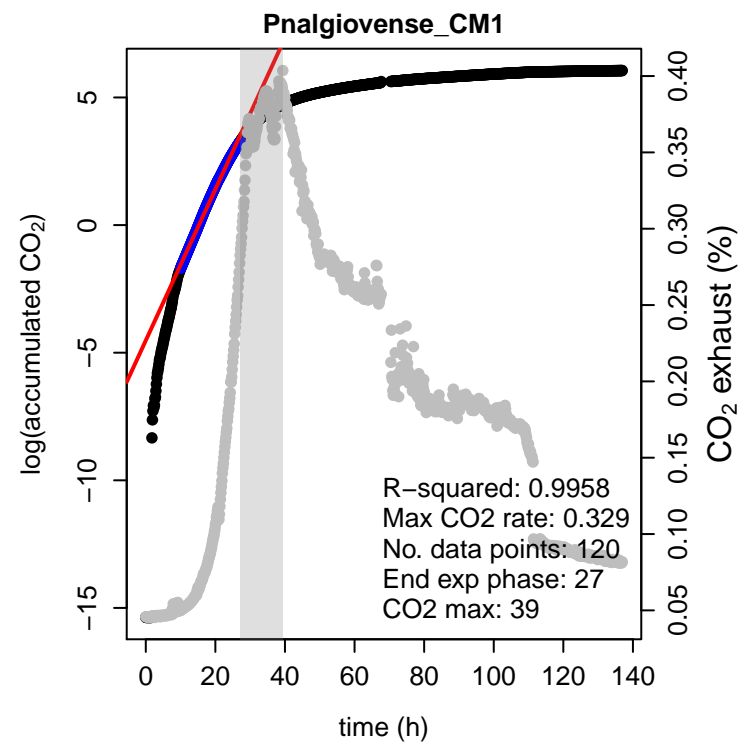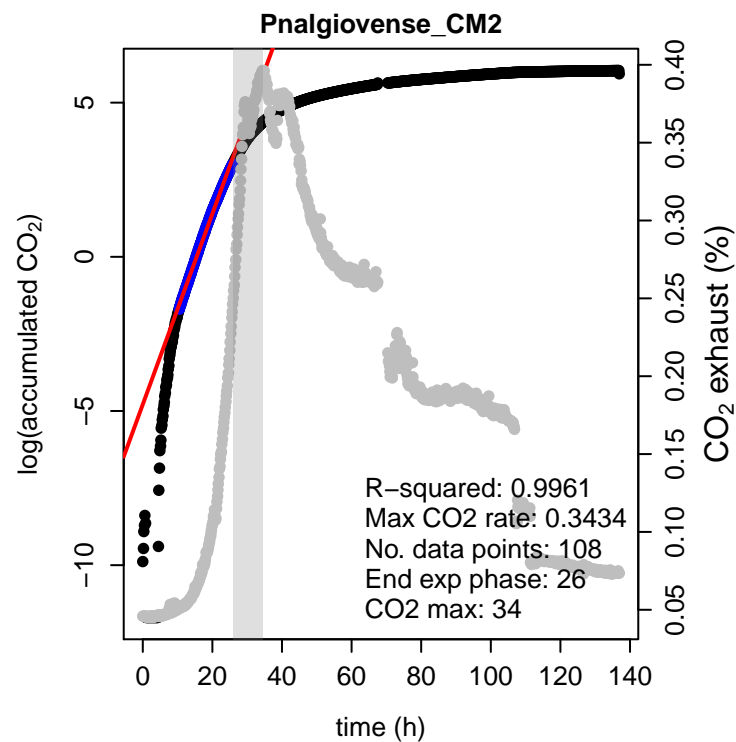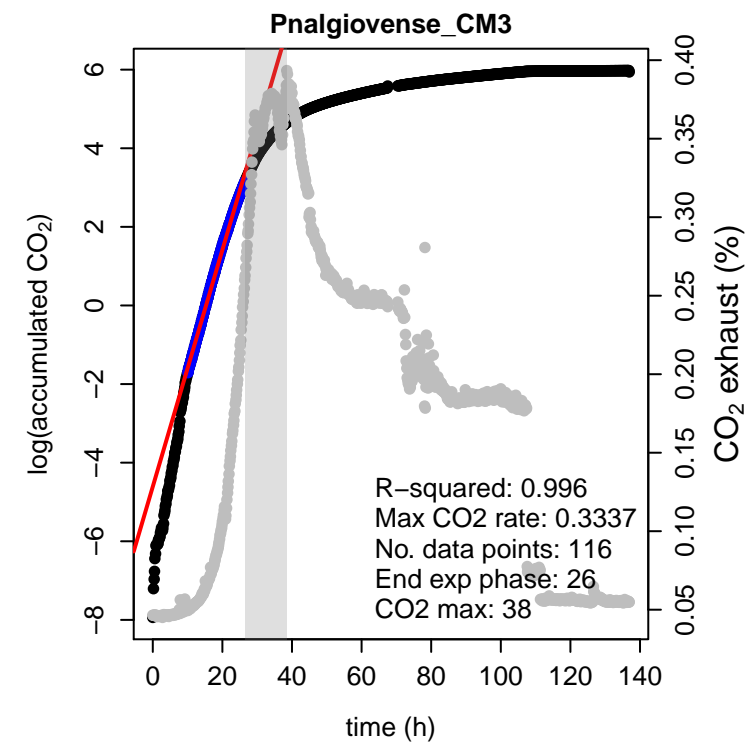

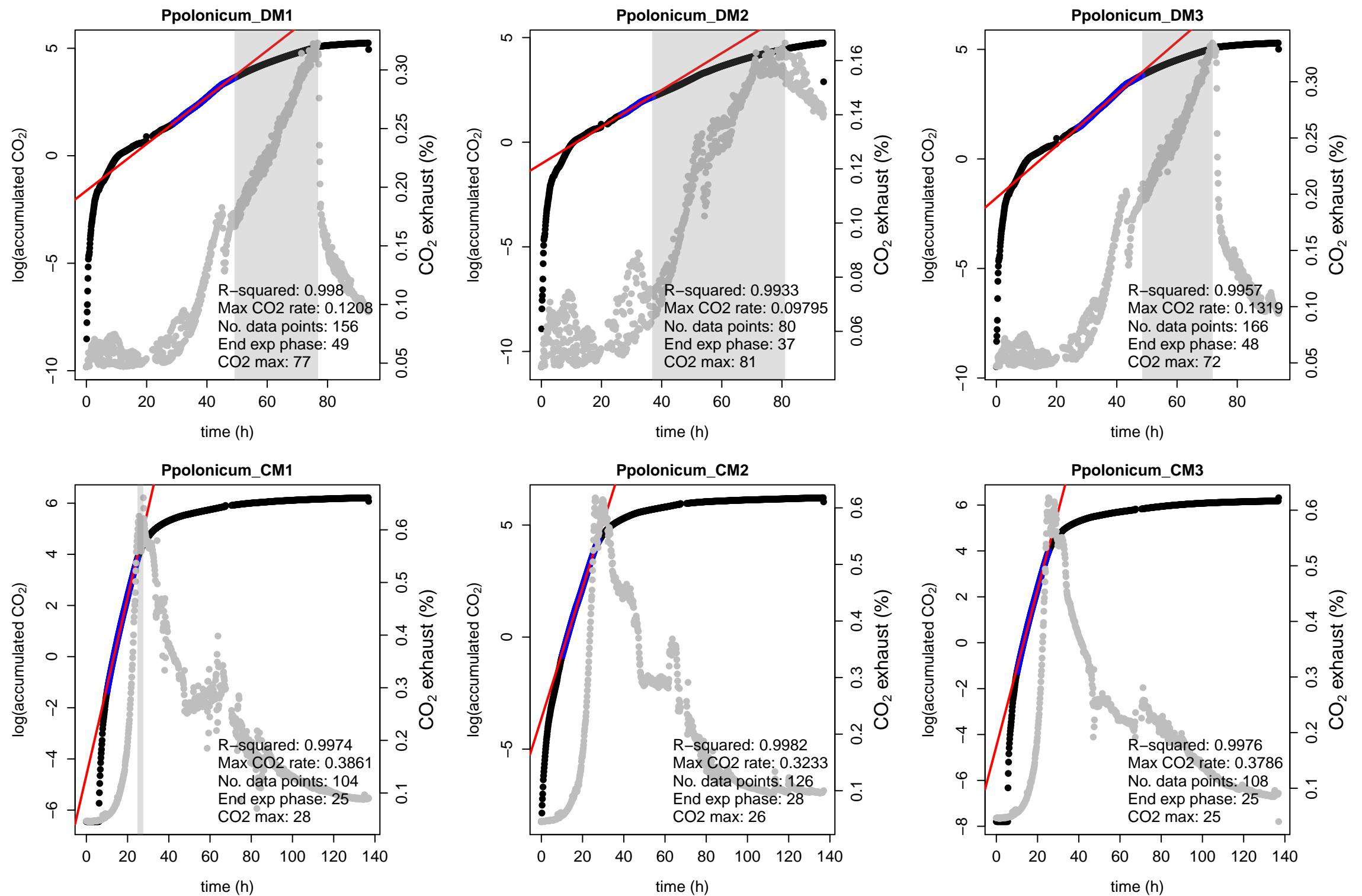

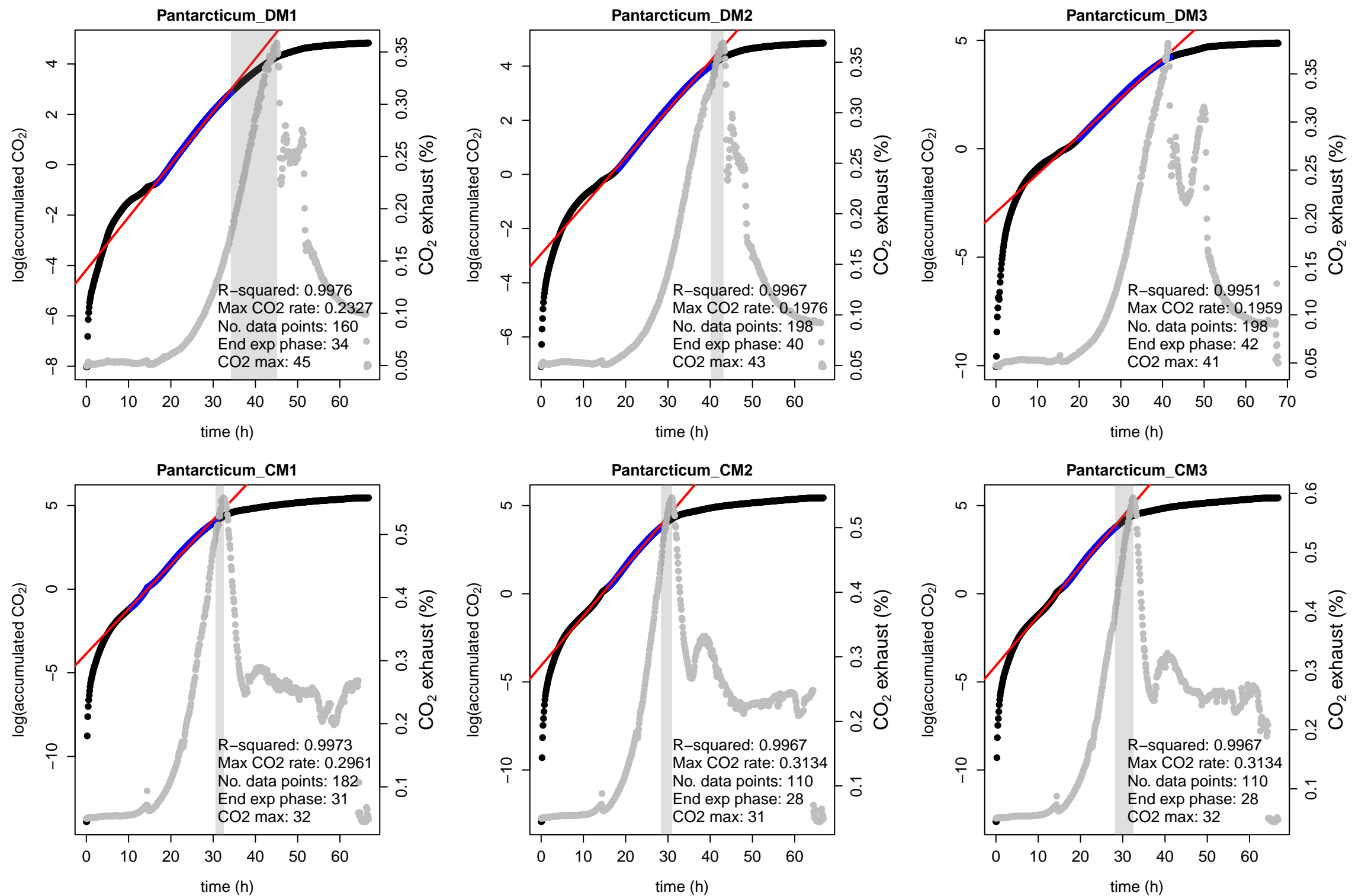

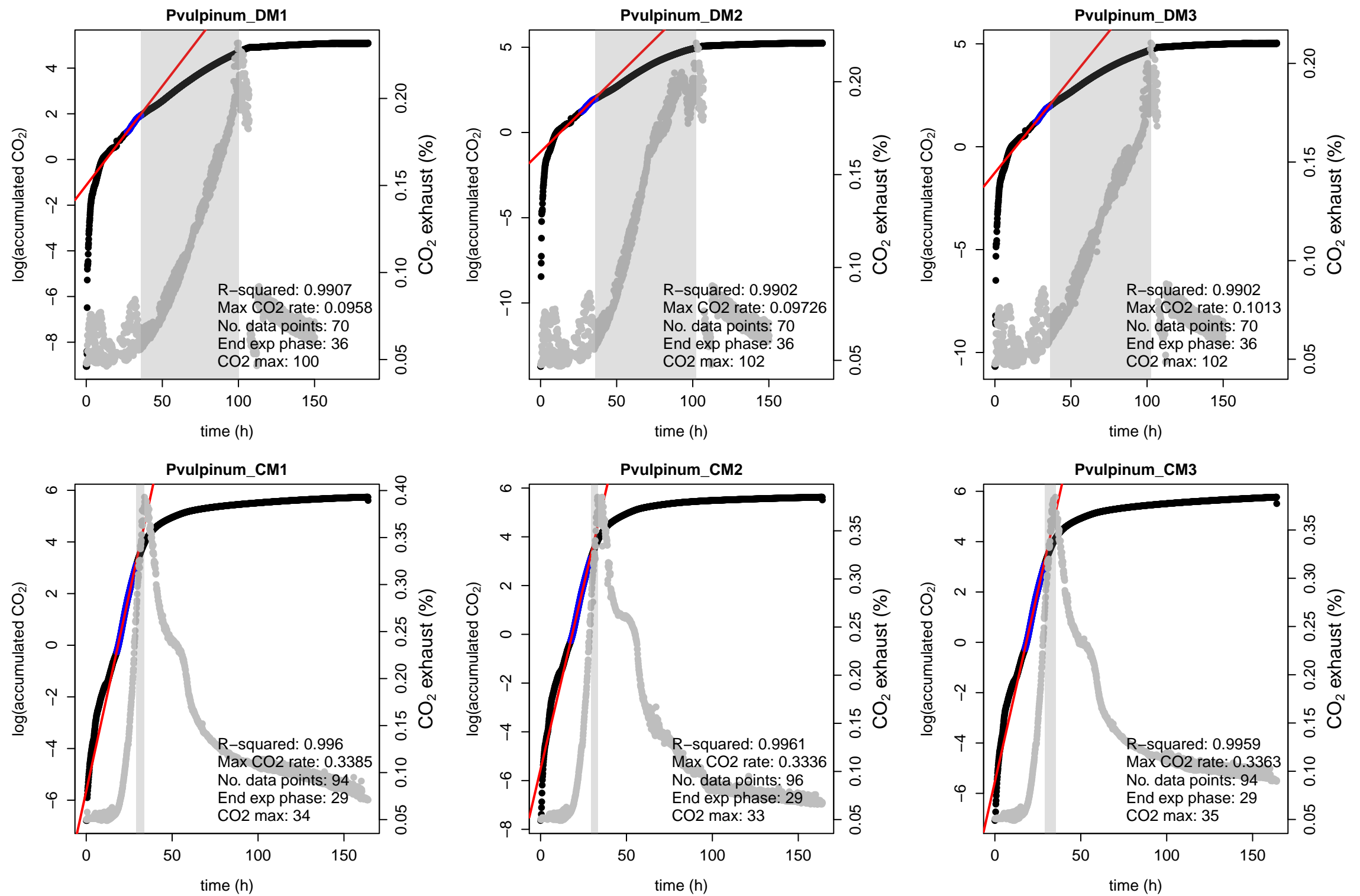

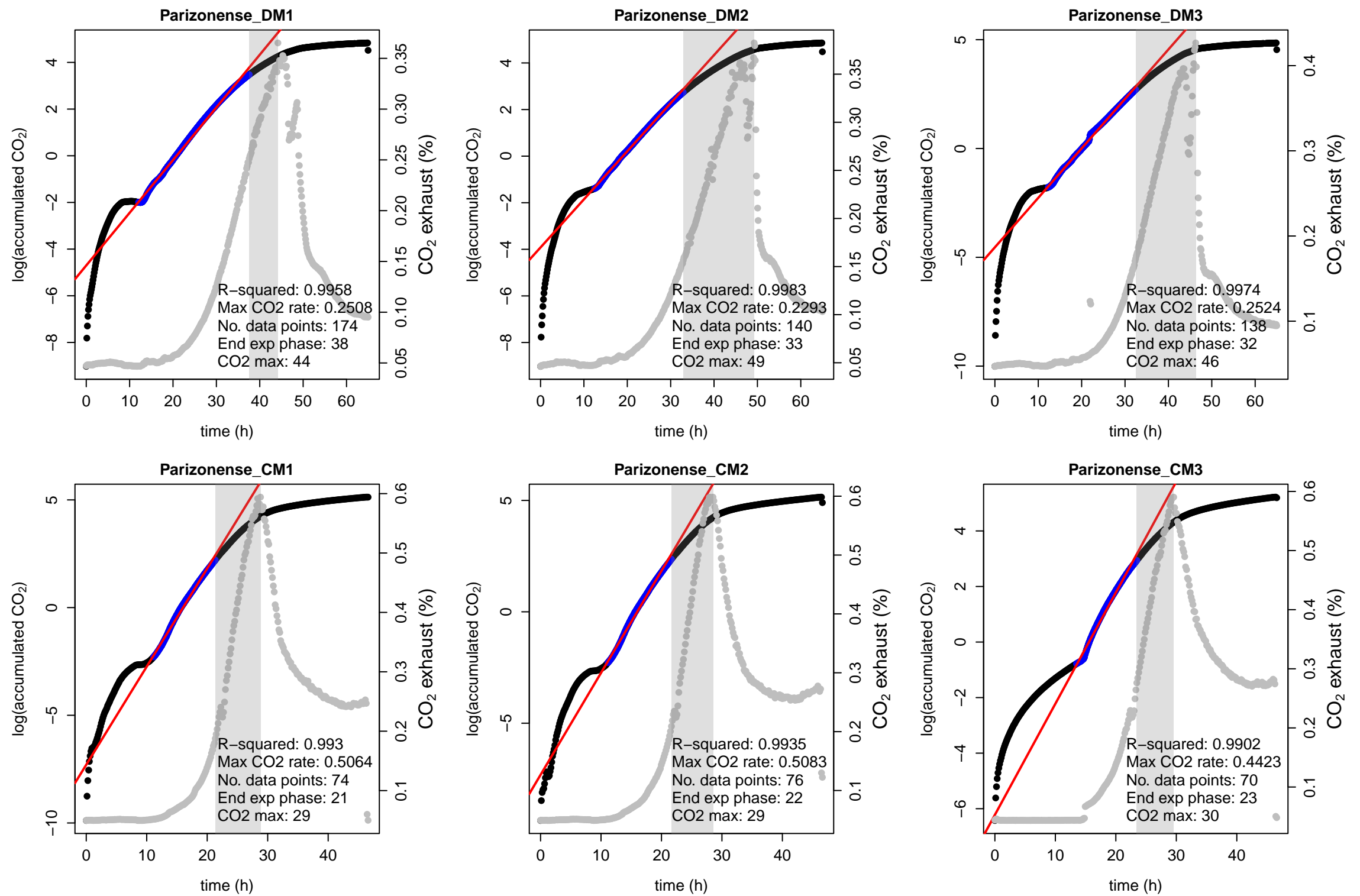

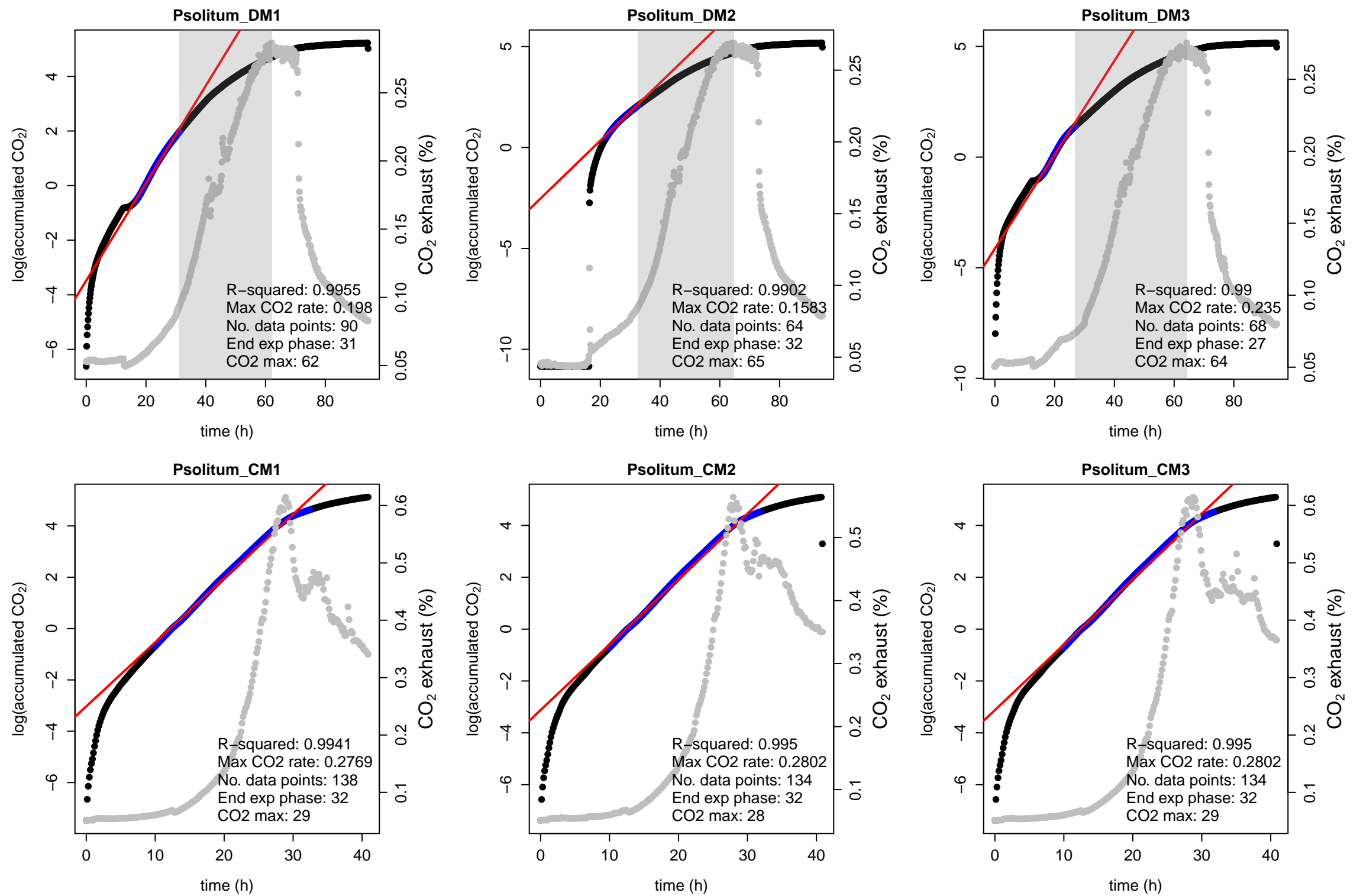

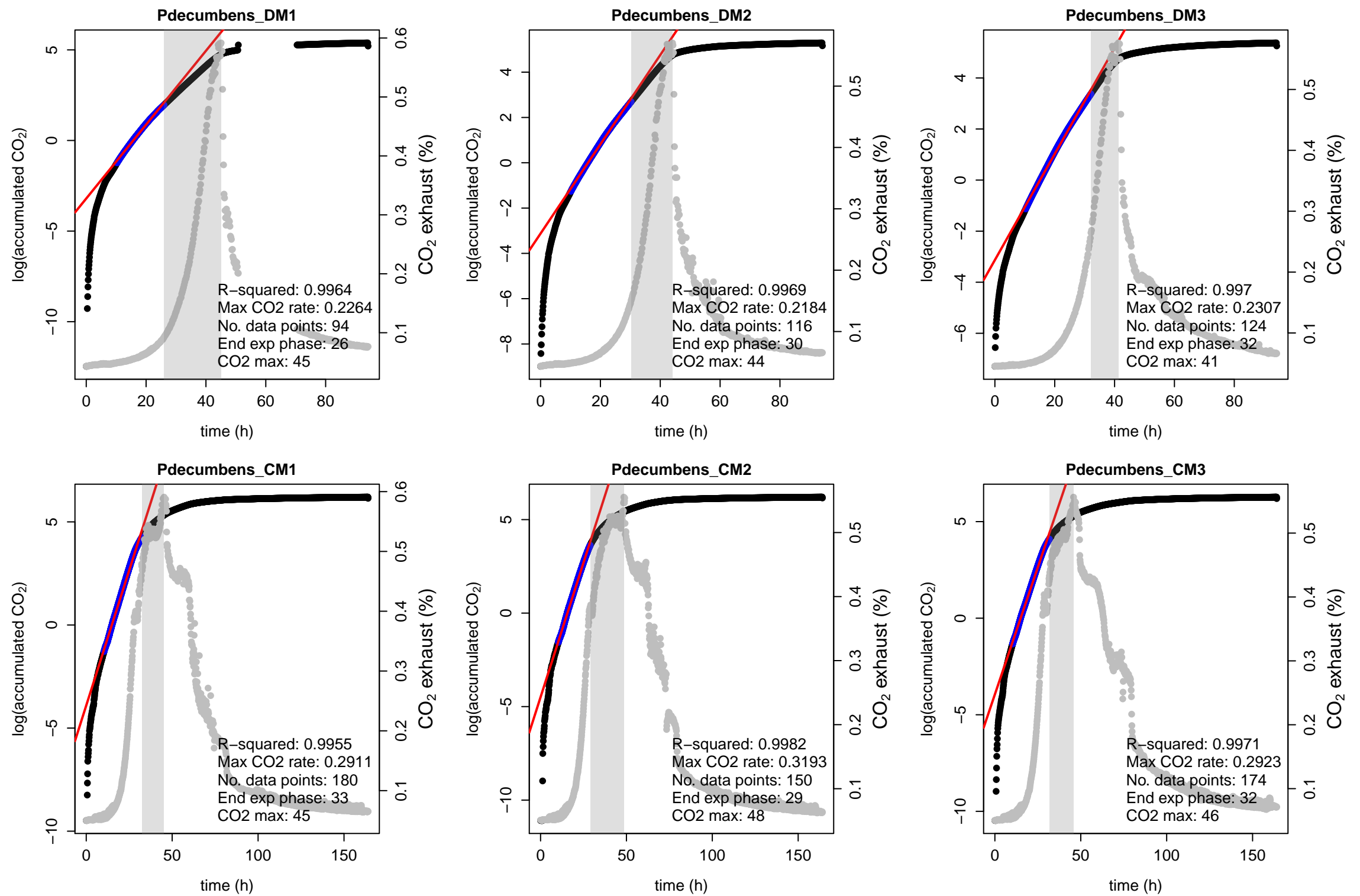

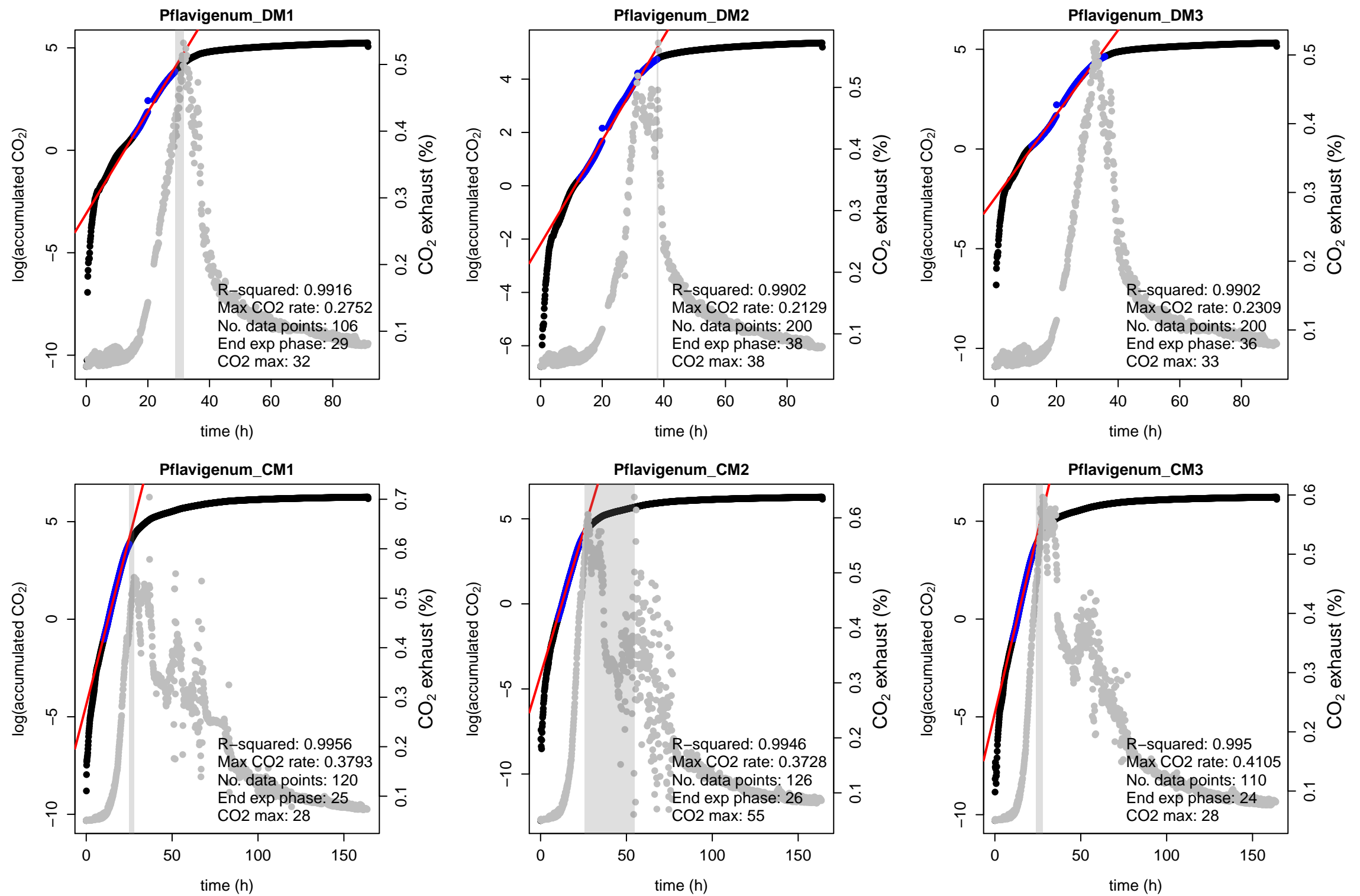

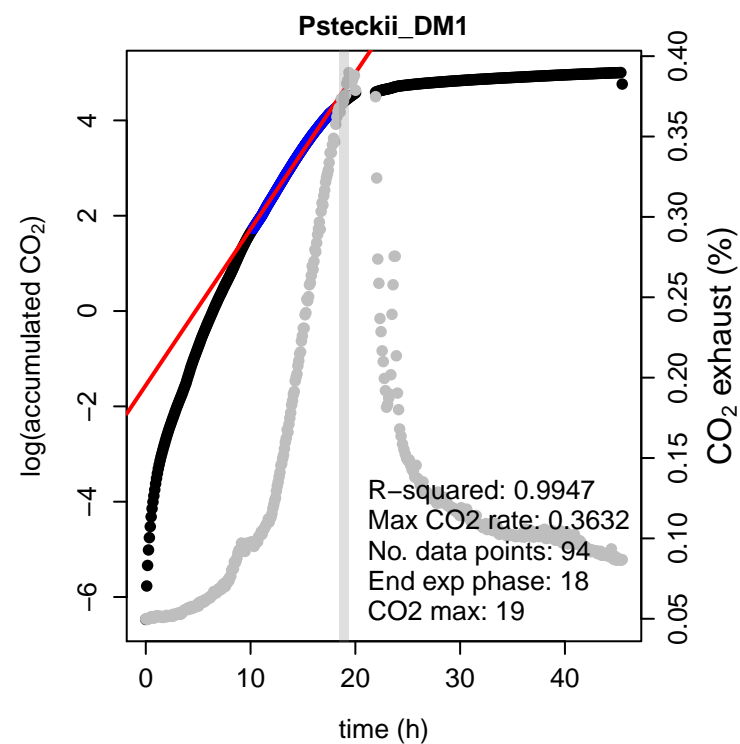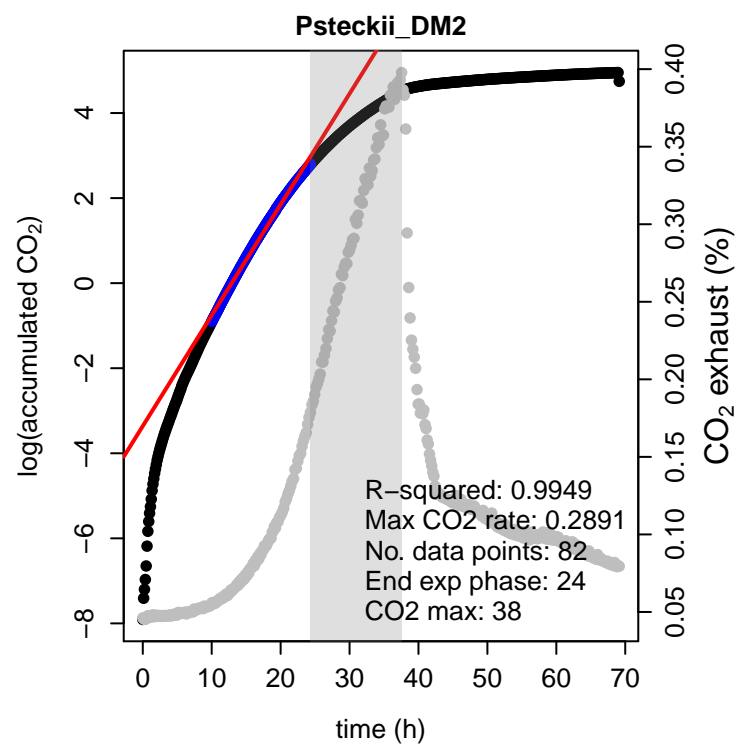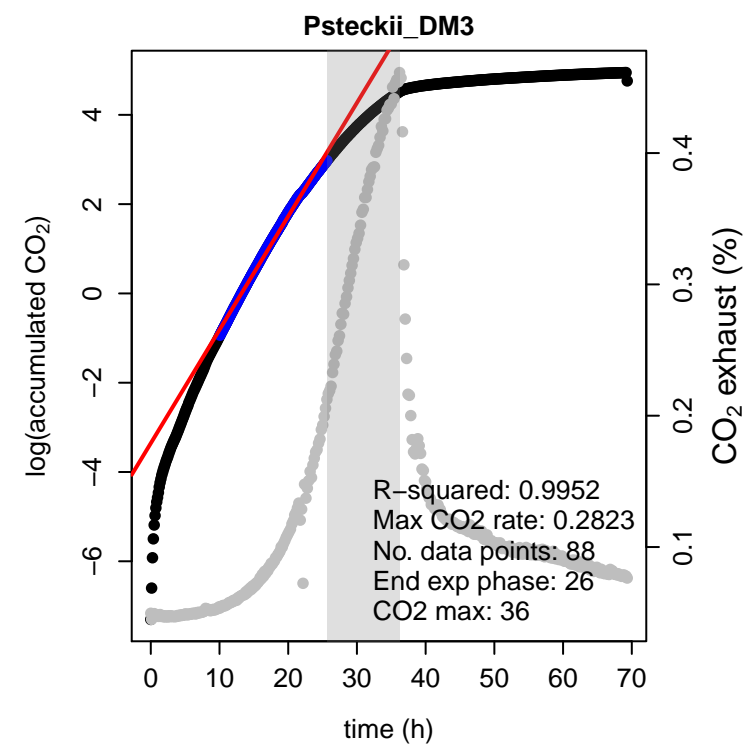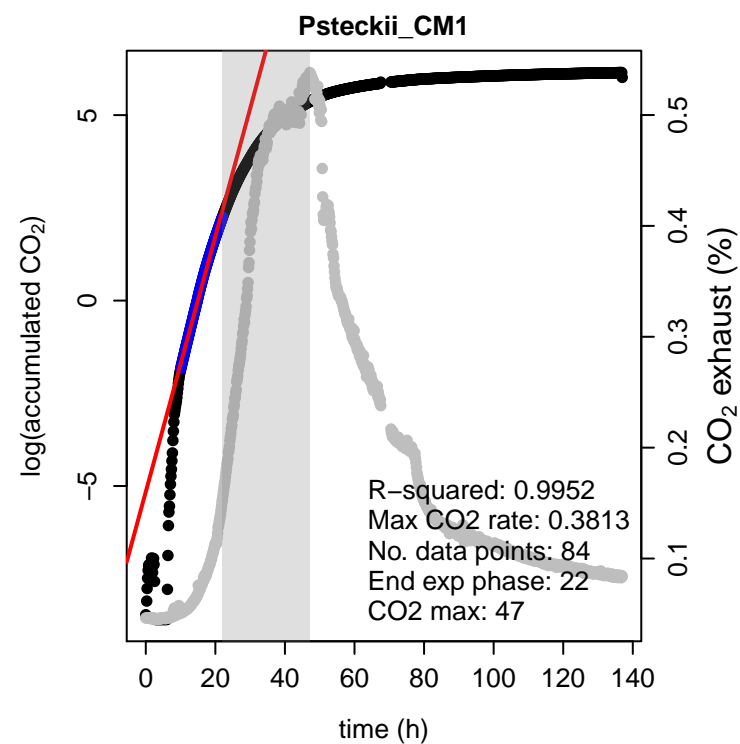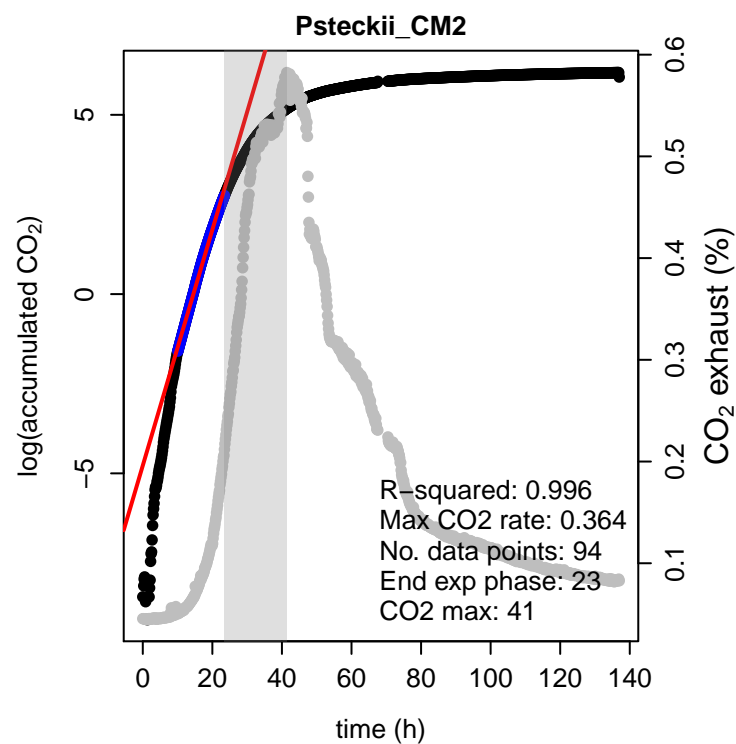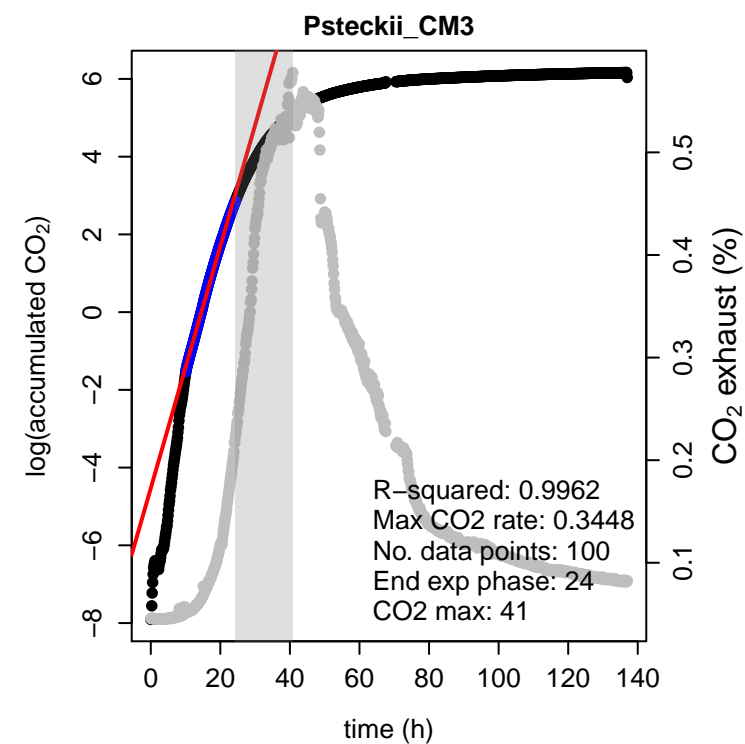

Supplement: Supplementary file 1 — Additional file 1. Physiological data. Physiological characteristics for triplicate fermentations of each species in DM and CM. Figures show the CO2 exhaust values and log value of the accumulated CO2. Furthermore a red line through the data points in exponential phase and highlighted in light grey the non-exponential phase; the time between start of non-exponential growth until the maximum CO2 off gas value. Additionally the R-squared value for the exponential phase, CO2 production rate, the number of data points in exponential phase, the end point (in hours) of the exponential phase and the end point of the non-exponential phase (in hours) are shown. [file 40694_2017_36_MOESM1_ESM.pdf]
